# Psychometric properties of the Persian version of the Emotion Regulation Questionnaire

**DOI:** 10.47626/2237-6089-2018-0106

**Published:** 2021-05-08

**Authors:** Ali Akbar Foroughi, Aliakbar Parvizifard, Kheirollah Sadeghi, Arash Parsa Moghadam

**Affiliations:** 1 Department of Clinical PsychologySchool of MedicineKermanshah University of Medical SciencesKermanshahIran Department of Clinical Psychology, School of Medicine, Kermanshah University of Medical Sciences, Kermanshah, Iran.; 2 Student Research CommitteeKermanshah University of Medical SciencesKermanshahIran Student Research Committee, Kermanshah University of Medical Sciences, Kermanshah, Iran.

**Keywords:** Factor analysis, Gross’s emotion regulation, reliability, validity

## Abstract

**Introduction:**

Gross’s Emotion Regulation Questionnaire is one of the most widely-used and valid questionnaires for assessing emotion regulation strategies. The validity and reliability of the Persian version have not been determined and data on its psychometric properties are not available to Iranian mental health researchers. The purpose of this study was to determine the psychometric properties of the Emotion Regulation Questionnaire in Iranian students.

**Methodology:**

In this cross-sectional study, 348 students (170 males and 178 females) were selected from Shahid Beheshti University of Medical Science and Tehran University of Medical Science. The following statistical procedures were conducted: correlation coefficients, factor analysis, Cronbach’s alpha, and independent *t *tests.

**Results:**

The results showed that men use suppression more than women (T = -2.62, p = 0.009). Cronbach’s alpha coefficients were 0.76 for the cognitive reappraisal sub-scale and 0.72 for the suppression sub-scale (excluding question 9). Six questions related to the cognitive reappraisal factor explained 30.97% of emotion regulation variance, and 3 questions related to the suppression factor explained 22.59% of emotion regulation variance. Overall, these factors explained 53.5% of emotion regulation variance. There were significant correlations between suppression and difficulties in emotion regulation, trait anxiety, and affective control. Furthermore, there was a significant correlation between cognitive reappraisal and the Five-Facet Mindfulness Questionnaire.

**Conclusion:**

The results indicate that the Persian version of the ERQ is a reliable and valid instrument that can be helpful for development of further important studies of emotional regulation.

## Introduction

Emotion is an individual’s overall, intense, and brief response to an unexpected event, accompanied by pleasant or unpleasant emotional states. Emotion has always been of interest to mental health researchers, for various reasons, including evolutionary function,^1^ social-communication,^2^ decision-making,^3^ and the important role it plays in mental health.^4^ In recent decades, there have been many advances in the field of emotion regulation, including scientific theories and studies. Hence, we have achieved a better understanding of the pathway of growth, neurology, genetic and environmental effects, and its relation to cognition.^5^ One of the most important issues in mental health is emotion regulation. Emotion regulation relates to a process in which individuals experience and express their emotions. According to Gross, the process of emotion regulation is further examined through cognitive reappraisal and expressive suppression, i.e. emotion regulation strategies that are activated at the beginning of an event or before it, and those that are activated after an event or an emotion. Gross believes that emotion regulation strategies do not represent the person’s positive or negative character, but rather are based on specific situations in the person’s life.^6^

Health professionals believe that problems with emotion regulation play a major role in maintenance and increase of mental disorders and maladaptive behaviors.^7^ Emotion regulation strategies play an essential role in mental health and psychiatric disorders such as depression,^8^ anxiety,^9^ borderline personality disorder^10,11^ and anorexia nervosa.^12^ Saxcena et al. found that difficulties in emotion regulation and use of dysfunctional emotion regulation strategies are factors that have a negative impact on mental health.^13-15^ In general, in most psychiatric disorders, there is at least one symptom that reflects impairment of emotions.^16^

Various instruments have been developed to measure the emotions. One of the most widely used instruments is Gross’s Emotion Regulation Questionnaire (ERQ). The ERQ is based on a theory-based approach and an emotion regulation model and has two sub-scales, cognitive reappraisal and expressive suppression. Cognitive reappraisal indicates that an individual makes an effort to change how he or she thinks about a situation in order to change its emotional impact and reappraise the initial perception, whereas expressive suppression is defined as a response-focused strategy.^17^ All items are answered on a 7-point Likert scale ranging from 1 (strongly disagree) to 7 (strongly agree), with higher scores representing higher usage of that strategy.

Gross & John, reported that the ERQ has a two-factor structure which means “reappraisal and suppression are two independent regulatory strategies that different individuals use to varying degrees.” Cronbach’s alphas were 0.79 for cognitive reappraisal and 0.73 for expressive suppression.^17^ Other studies have also shown that ERQ has good validity and reliability. For example in a study by Eldeleklioğlu & Eroğlu, Cronbach’s alphas were 0.78 for cognitive reappraisal and 0.73 for expressive suppression.^18^ Furthermore, in a study conducted by Enebrink et al., Cronbach’s alpha coefficients were 0.81 for cognitive reappraisal and 0.73 for expressive suppression.^19^ Similarly, in Balzarotti et al., Cronbach’s alpha coefficients were 0.84 for cognitive reappraisal and 0.72 for expressive suppression.^20^

However, no studies have determined the validity and reliability of the Persian version of the ERQ and no psychometric properties have been available to Iranian mental health researchers so far. The purpose of the present study was to evaluate the internal consistency and factor structure of an Iranian adaptation of the ERQ.

## Methodology

Participants were selected from among the undergraduate, postgraduate, and PhD students at Shahid Beheshti University of Medical Sciences and Tehran University of Medical Sciences. Determining an appropriate sample size for structural equation modeling is a seminal element of factor analysis. Klein believes that 10 or 20 samples are necessary for each variable in exploratory factor analysis, but a sample size of at least 200 can be defended.^21^ The sample selected comprised 348 students (170 males and 178 females) who were chosen from the aforementioned universities. These participants were selected using a convenience sampling method. The Inclusion criterion was Persian as a native language and exclusion criteria were “having a severe psychiatric disorder and unwillingness to participate in research.”

The ERQ was separately translated into Persian by two PhD students in clinical psychology, and afterwards a PhD professor in clinical psychology rectified discrepancies in the translations. In the next step, two English-language experts were asked to translate them back into the original language. The translated texts were compared with the original text and any problems were investigated, including the structures of translated sentences. In the next step, the scale was administered to a sample of 20 participants and problems such as ambiguity and incomprehensibility of a few Persian sentences were rectified. After taking these steps, the questionnaire was finally utilized. The study was designed according to the Declaration of Helsinki and approved by the Ethics Committee at Shahid Beheshti University of Medical Sciences (IR.SBMU.SM.REC.1394.181) and all participants gave their consent to take part in the study, signing the consent form.

### Instruments

#### Five-Facet Mindfulness Questionnaire (FFMQ)

The FFMQ is used to measure the subjective view of one’s mindfulness. The FFMQ evaluates five facets of mindfulness: observing, describing, acting with awareness, non-reactivity (to inner experience), and non-judging (of inner experience). It was developed by Baer et al.^22^ The 39 items on the FFMQ are rated on a 5-point Likert scale ranging from 1 (never) to 5 (always true). FFMQ scores are obtained by summing up the scores of the individual items. FFMQ scores range from 8 to 40, with higher scores representing more mindfulness.

The FFMQ has adequate internal consistency and the alpha coefficients of its sub-scales have been reported as follows: 0.91 for describing, 0.83 for observing, 0.87 for acting with awareness, 0.75 for non-reactivity, and 0.87 for non-judgmental inner experience.^18^ The reliability and validity of this test in Iranian samples was desirable (alpha ranged from 0.55 to 0.83). There were positive and significant correlations between the five personality factors and the five dimensions of mindfulness, with the exception of neuroticism.^23^ Furthermore, positive correlations were observed between psychological well-being and mindfulness sub-scales, while negative correlations were observed between mindfulness sub-scales and all the symptoms of the SCL-25.

#### State-Trait Anxiety Inventory (STAI)

The STAI was designed to measure anxiety in the form of state and trait. In this study, only the trait anxiety part was used, which has 20 items and scores ranging from 20 to 80. The Cronbach’s alpha for trait anxiety is equal to 0.9.^24^ In Gholami Booreng’s study, the Cronbach’s alpha coefficient was also reported to be 0.9.^25^

#### Affective Control Scale (ACS)

The ACS measures people’s control over their emotions and includes 42 questions with four sub-scales that measure fears of emotion and attempt to control emotional experiences.^26^ The instrument’s sub-scales include four fears: fear of anxiety, of depression, of anger, and of positive emotion. The ACS is a self-report questionnaire rated on a 7-point Likert scale. It assesses attention control and attention shifting. The responses for items number 4, 9, 12, 16, 17, 18, 21, 22, 27, 30, 31, and 38 should be inverted. Its internal and test-retest reliabilities were found to be 0.94, 0.78 respectively. Internal and test-retest reliability indices for the fear of anger, fear of depression, fear of anxiety, and fear of positive emotion sub-scales were estimated as follows: 0.72 and 0.73; 0.91 and 0.76; 0.77 and 0.89; and 0.64 and 0.84. In Iran, a study by Dehesh reported an overall Cronbach’s alpha of 0.84, and Cronbach’s alphas of 0.53, 0.60, 0.76, and 0.64 for the sub-scales fear of anger, fear of emotion, fear of depression, and fear of anxiety.^27^

#### Difficulty in Regulation of Emotion Scale (DRES)

The DRES consists of 36 items.^10^ DRES items are rated on a 5-point Likert scale ranging from 1 (almost never) to 5 (almost always). Higher scores indicate greater difficulty with emotion regulation. The results of exploratory factor analysis in the Iranian sample revealed eight factors, from which six factors coincided with those of the original version and two other factors were excluded. Furthermore, there was an internal correlation with Beck’s depression and anxiety questionnaire.^28^

## Statistical analyses

### Kaiser-Meyer-Olkin (KMO)

The KMO index is a sampling coefficient index that indicates the proportion of variance among the variables that might be caused by underlying factors. This index ranges from 0 to 1. When the value approaches 1, the sampling of the data is adequate for performing factor analysis, otherwise (usually if KMO is less than 0.5) the factor analysis probably falls short of validity.^29^

### Bartlett’s test

Another method for determining the suitability of data is Bartlett’s test. This test examines the hypothesis that the observed correlation matrix belongs to a group with nonrelated variables. For a factor model to be useful and meaningful, the variables need to be correlated together. Small significance level values (< 0.05) indicate that a factor analysis appears to be appropriate for the data tested. If the significance level is less than 0.05, the factor analysis can coordinate with the data, since the assumption of correlation matrix unity is rejected.^30^

### Confirmatory factor analysis

Confirmatory factor analysis (CFA) is a statistical method used to investigate the factor structure of a set of observed variables. CFA let the researcher test the hypothesis that a relationship between observed variables and their underlying latent constructs exists. In confirmatory research (also known as hypothesis testing), the researcher has a good specific idea about the relationships between the variables under investigation and the researcher attempts to find whether a theory, which is specified as a hypothesis, is supported by data.^29^

## Results

Twenty-four participants were excluded due to missing information needed for the final analysis. Descriptive analysis of the data collected on the participants is shown in Table 1, and the means and standard deviations for the other questionnaires are listed in Table 2.

**Table 1:** Descriptive data on participants

Faculty	n (%)	Age, mean (SD)	Sex
Medicine	156 (44.8)	19.5 (1.84)	F: 81, M: 75
Dentistry	84 (24.1)	20.07 (2.55)	F: 43, M: 41
Pharmacy	14 (4)	23.07 (5.66)	F: 8, M: 6
Paramedicine	86 (24.7)	23.38 (5.25)	F: 42, M: 44
Basic Sciences	8 (2.3)	31.50 (4.98)	F: 4, M: 4
Total	348	23.50 (4.05)	F: 178, M: 170

F = female; M = male; SD = standard deviation.

**Table 2:** Mean, standard deviation, and minimum and maximum scores of questionnaires

Measures	FFMQ	DRES	STAI	ACS
Mean	98.3	37.8	41.6	73.3
Standard deviation	12.6	18.7	9.6	17
Minimum	68	0	20	25
Maximum	143	88	74	114

ACS = Affective Control Scale; DRES = Difficulties in Regulation of Emotion Scale; FFMQ = Five Facet Mindfulness Questionnaire; STAI = The State-Trait Anxiety Inventory.

The independent *t* test for the ERQ subscales showed that men used suppression more than women, and this difference was statistically significant (p = 0.009, T = -2.62). Additionally, women used cognitive reappraisal more than men, but this difference was not statistically significant (T = 1.31, p = 0.759).

The KMO index and Bartlett’s test of sphericity showed the sample was adequate for factor analysis (approximate chi-square = 0.734; df = 36; Sig = 0.001; sample size is greater than 0.5). Furthermore, the significance level is less than 0.05. We used the FFMQ, ACS, DRES, and TRAIT scales to evaluate the divergent and convergent validity of the ERQ. Pearson correlation coefficients were calculated and these results are presented in Table 4. Cohen determined that a correlation coefficient of 0.10 represents a weak or small association, a correlation coefficient of 0.30 is considered a moderate correlation, and a correlation coefficient of 0.50 or larger represents a strong or large correlation.^31^

**Table 4:** Correlations for the Emotion Regulation Questionnaire subscales and other scales

Measures	FFMQ	DRES	STAI	ACS	Suppression	Reappraisal
FFMQ	-					
DRES	-0.68*	-				
TRAIT	-0.54*	0.56*	-			
ACS	-0.52*	0.57*	0.54*	-		
Expressive suppression	-0.28*	0.25*	0.21*	0.09	-	
Cognitive reappraisal	0.11^†^	-0.11^†^	-0.24*	-0.06	-0.14*	-

ACS = Affective Control Scale; DRES = Difficulties in Regulation of Emotion Scale; FFMQ = Five-Facet Mindfulness Questionnaire; STAI= State-Trait Anxiety Inventory;.

* p < 0.01; ^†^ p < 0.05.

As shown in Table 4, the correlations between expressive suppression and the three variables DRES, TRAIT, and FFMQ are negative. Additionally, there was a positive and significant relationship between cognitive reappraisal and FFMQ, and negative and significant correlations with ACS, DRES, and TRAIT, indicating adequate divergent validity of this sub-scale.

As shown in Table 5, Cronbach’s alpha coefficients were 0.76 for cognitive reappraisal and 0.72 for expressive suppression (after elimination of item 9). These values were both greater than 0.7, indicating the questionnaire is reliable. Factor analysis results showed that 6 of the 10 questions on the ERQ were loaded onto the cognitive reappraisal factor (30.97%) and 3 questions (2, 4, and 6) were loaded onto expressive suppression (22.95%). Question 9 was omitted because it had high factor loadings for both cognitive reappraisal and expressive suppression factors.

**Table 5:** Factor loadings, eigenvalues, and variances of the Emotion Regulation Questionnaire subscales

Items (questions)	Reappraisal	Suppression
1. I control my emotions by changing the way I think about the situation I’m in.	0.65	
2. When I want to feel less negative emotion, I change the way I’m thinking about the situation.		0.78
3. When I want to feel more positive emotion, I change the way I’m thinking about the situation.	0.72	
4. When I want to feel more positive emotion (such as joy or amusement), I change what I’m thinking about.		0.81
5. When I want to feel less negative emotion (such as sadness or anger), I change what I’m thinking about.	0.69	
6. When I’m faced with a stressful situation, I make myself think about it in a way that helps me stay calm.		0.78
7. I control my emotions by not expressing them.	0.66	
8. When I am feeling negative emotions, I make sure not to express them.	0.60	
10. When I am feeling positive emotions, I am careful not to express them.	0.74	
Cronbach’s alpha coefficients	0.76	0.72
Eigenvalues	2.78	2.03
Factor variances	30.97	22.59
Total variance	53.56	

## Discussion

The results of the present study are in line with previous studies. The present study shows that men used suppression more than women did. Significant correlations were observed between the TRAIT, DRES, and FFMQ questionnaires and the cognitive reappraisal and expressive suppression ERQ subscales. The Cronbach’s alpha coefficients for cognitive reappraisal and for expressive suppression were adequate and indicated good internal consistency. Factor analysis showed that 6 out of the 10 items on the ERQ items loaded onto the cognitive reappraisal factor and 3 items loaded onto expressive suppression.

The results of the present study showed that men used suppression more than women, and this difference was statistically significant. Furthermore, women used cognitive reappraisal more than men, but this difference was not statistically significant. This finding is consistent with the findings of Gross et al.,^17^ Balzarotti et al.,^20^ Enebrink et al.,^19^ and Wiltink et al.,^32^ whereas research conducted by Mehri and Kazarian did not find a difference between men and women in use of suppression.^33^ This discrepancy could be explained by taking account of different variables such as research sample and different countries’ cultures. In studies by Gross et al. and by Wiltink and Enebrink, the research sample included university students, but the research samples in the studies by Balzarotti et al. and Eldeleklioğlu & Eroğlu, included members of the general community.^18, 20^

The results of the present study denoted that the correlations between suppression items and the DRES and STAI were significantly positive, which demonstrates the convergent validity of the suppression sub-scale (Table 4). Moreover, the negative correlations of suppression items with the FFMQ indicated appropriate divergent validity. A significant positive relationship between cognitive reappraisals and the FFMQ indicated adequate convergent validity (Table 4). The significant negative correlations between the ACS and the DRES and the STAI proved that the divergent validity of cognitive reappraisals sub-scale was adequate (Table 4). The findings of the present research are in line with results published by Wiltink et al. and by Abler & Kessler. Wiltink et al. showed that the negative relationship between cognitive reappraisal and anxiety that was reported denoted a significant relationship between repression and anxiety.^32^ Abler & Kessler found a significant relationship between suppression and anxiety.^34^ To explain this report, it can be stated that mindfulness is described as non-judgmental and momentary attention to current experience. Mindfulness has psychological effects such as decreases in psychological symptoms and emotional dysfunction and improvement of behavioral regulation. In addition, mindfulness also increases people’s ability to tolerate negative emotions and prepares them for well-adjusted behaviors in different situations. The present study indicated a significant correlation between expressive suppression and cognitive reappraisal. These findings are not in line with the study by Christos, Loannidis, and Siegling, which demonstrated a weak correlation between cognitive reappraisal and expressive suppression, and found that these two scales were independent of each other.^35^ Addressing these discrepancies, the authors consider that cultural differences between different societies are influential factors.

The results of this study showed that Cronbach’s alpha coefficients for cognitive reappraisal and expressive suppression (with the elimination of item 9) were 0.76 and 0.72 respectively. Since both values are greater than 0.7, they attest to the questionnaire’s reliability. This finding is in line with Gross & John’s study, in which Cronbach’s alphas for cognitive reappraisal and suppression were 0.79 and 0.73 respectively. In Eldeleklioğlu & Eroğlu’s study, Cronbach’s alphas were 0.78 for cognitive reappraisal and 0.73 for expressive suppression.^18^ In a study conducted by Enebrink et al., Cronbach’s alphas for cognitive reappraisal and expressive suppression were 0.81 and 0.73 respectively,^19^ and Balzarotti et al. reported Cronbach’s alphas for cognitive reappraisal and expressive suppression of 0.84 and 0.73 respectively.^20^

The factor analysis in this study showed that 6 out of 10 ERQ items loaded onto cognitive reappraisal and 3 items (items 2, 4, and 6) loaded onto expressive suppression (Table 5). Item 9 was excluded because of its high factor loadings onto both the cognitive reappraisal and expressive suppression factors. Six items relevant to the cognitive reappraisal factor explained 30.97 percent of emotion regulation variance and 3 items pertaining to the suppression factor explained 22.95 percent of emotional regulation variance. Together, these two factors explain 53.5 percent of total variance in emotion regulation (Table 5). These results are in line with the findings of Enebrink et al., who reported that there was a correlation between the suppression and cognitive reappraisal sub-scales.^19^ Enebrink et al. showed that modification indices (MI) suggested that the model would achieve a better fit by maintaining items 4 and 9 as linked to cognitive reappraisal, even though both of these items are obvious examples of suppression. Furthermore, MI suggested a path between cognitive reappraisal and item 9 on the ERQ along with two paths from expressive suppression to items 8 and 10. Moreover, the results of the study by Wiltink et al. showed that item 9 had substantial loading onto cognitive reappraisal as well.^32^

## Limitations

The findings of the convenience sampling method cannot be generalized to other populations. Moreover, since the participants were selected from undergraduate, postgraduate, and PhD students at Shahid Beheshti University of Medical Sciences and Tehran University of Medical Sciences, the findings cannot be generalized to the general population including children and adults.

## Conclusion

The results of confirmatory factor analysis showed that the ERQ had good psychometric properties and that its Cronbach’s alpha coefficient was adequate. Convergent and divergent validity were observed between TRAIT, DRES, FFMQ, DERS questionnaires and ERQ sub-scales. Therefore, the Persian version of the ERQ is a reliable and valid instrument that has consistency and should be useful for development of further important studies on emotional regulation.

**Table 3:** Kaiser-Meyer-Olkin measure of sampling adequacy and Bartlett’s test of sphericity

	Kaiser-Meyer-Olkin measure of sampling adequacy	
Bartlett’s test of sphericity	Approx. chi-square	0.734
	df	36
	Sig	0.001

df = degrees of freedom.
